# Reversible end-to-end assembly of selectively functionalized gold nanorods by light-responsive arylazopyrazole–cyclodextrin interaction

**DOI:** 10.3762/bjoc.15.140

**Published:** 2019-06-26

**Authors:** Maximilian Niehues, Patricia Tegeder, Bart Jan Ravoo

**Affiliations:** 1 Organic Chemistry Institute and Center for Soft Nanoscience, Westfälische Wilhelms-Universität Münster, Corrensstraße 40, D-48149, Germany

**Keywords:** cyclodextrins, gold nanorods, host–guest chemistry, light-responsive materials, molecular switches, self-assembly

## Abstract

We propose a two-step ligand exchange for the selective end-functionalization of gold nanorods (AuNR) by thiolated cyclodextrin (CD) host molecules. As a result of the complete removal of the precursor capping agent cetyltrimethylammonium bromide (CTAB) by a tetraethylene glycol derivative, competitive binding to the host cavity was prevented, and reversible, light-responsive assembly and disassembly of the AuNR could be induced by host–guest interaction of CD on the nanorods and a photoswitchable arylazopyrazole cross-linker in aqueous solution. The end-to-end assembly of AuNR could be effectively controlled by irradiation with UV and visible light, respectively, over four cycles. By the introduction of AAP, previous disassembly limitations based on the photostationary states of azobenzenes could be solved. The combination photoresponsive interaction and selectively end-functionalized nanoparticles shows significant potential in the reversible self-assembly of inorganic–organic hybrid nanomaterials.

## Introduction

Metallic nanomaterials have received intense and interdisciplinary interest due to their unique optical [1], electronic [2–3] and sensing properties [4–5]. In particular, noble metal nanoparticles of sizes smaller than the wavelength of the incident light show interesting optical behavior as a result of collective oscillations of the valence electrons. This leads to surface plasmon resonance (SPR), which is highly dependent on size, shape and chemical environment giving rise to different SPR band wavelengths [6–8]. Especially gold nanorods (AuNR) are of interest because of their good synthetic availability and their unique optical properties. Due to their anisotropy AuNR possess a transversal SPR (TSPR) band in the visible and a longitudinal SPR (LSPR) band that can reach up to the near infrared (NIR) region [9]. The LSPR band can be tuned over a wider wavelength range than for isotropic nanoparticles by varying the aspect ratio of the AuNR or by the assembly of multiple AuNR into linear clusters [10]. These linear aggregates can be realized more or less efficiently through various approaches based on supramolecular interactions like metal–metal and π–π interactions [11], DNA mediated [12] or by host–guest chemistry [13]. Most of these approaches require selective functionalization of the ends of the AuNR and take advantage of the different ligand exchange kinetics of CTAB on the ends and the side of the particles [14–15]. CTAB serves as a capping agent in AuNR synthesis by the seed-mediated growth process and is essential for the anisotropic growth. Due to the different crystallographic environments on the particle surface, weaker packing and binding forces enable the preferential exchange of ligands on the facets at the ends [16]. Removal of CTAB on the side facets is relatively slow and requires a higher ligand concentration. However, for biotechnological applications it might be advantageous to remove CTAB from the complete surface because of its cell toxicity [17], hence strategies for replacing this coating are desirable.

Host–guest chemistry is a supramolecular interaction that is tailor-made for self-assembly due to its lock–key mechanism and has been applied in our and other groups to various nanoparticle systems [18–24]. The host–guest interaction can be responsive to external stimuli such as redox, pH or light, with the latter being the most desirable for assembling nanoparticles by virtue of its noninvasive nature [25]. Prominent light-responsive guest molecules are azobenzenes that form inclusion complexes with α- or β-CD exclusively in the *trans* configuration, not in the *cis* configuration [26]. This light-responsive interaction has been recently applied by Ma et al. for the end-to-end assembly of AuNR [27]. However, the system showed some limitations as the assembly was only achieved when the CD ligand and the divalent azobenzene linker were premixed to generate the host–guest complex. This solution was then added to the AuNR leading to a ligand exchange preferentially at the ends and therefore to the linear arrangement of AuNR. If the AuNR were first end-functionalized with the CD, no assembly could be observed after addition of the divalent azobenzene linker. Moreover, the assemblies could only once be disassembled by the combination of UV irradiation and physical forces by sonication. The light-induced back-isomerization of azobenzenes did not form similar end-to-end aggregates. Ma et al. suggested that the host–guest interaction is not strong enough to assemble the particles if not preformed. Yet, this is not consistent with publications that report photoswitchable azobenzene–CD interaction on surfaces [28], in hydrogels [29], the assembly of CD vesicles [30] or the assembly of Au nanoparticles [31].

It is our hypothesis that CTAB, which serves as surfactant and is essential in the synthesis of AuNR in seed-mediated growth processes, acts as a competitive binder to the CD cavity and prevents the reversible self-assembly. In fact, CTAB has a more than 20-fold higher binding constant to β-CD (6.5 × 10^4^ M^−1^) [32] than unmodified azobenzenes (2.5 × 10^3^ M^−1^) [33]. Therefore, the competitive binding of CTAB to β-CD has been applied previously, e.g., in sensing applications [34] or to enhance the water solubility of a cyclodextrin polymer [35]. On account of the experimental conditions that lead to a CTAB double layer formation around the AuNR, a significant amount of unbound CTAB may remain in solution and interact with host molecules. Furthermore, the light-responsive dispersion of the AuNR was only possible by the combination of irradiation and sonication, whilst some dimers could not be fully dispersed. Possibly this observation can be explained from the limited photostationary states (PSS) of azobenzenes (*E* – *Z*: 80%, *Z* – *E*: 70%) [36].

Arylazopyrazoles (AAP) are a new class of molecular switches introduced by Fuchter et al. with excellent photophysical properties like nearly quantitative isomerization, straight forward synthesis in excellent yields and very long *Z*-isomer half-life times up to 1000 days due to less steric repulsion [37]. In previous reports we could show that the AAP guest inclusion properties to β-CD are comparable to azobenzenes leading due to the superior photophysical properties to fully reversible supramolecular systems, which showed limited feasibility with azobenzenes [20,38–39].

**Scheme 1:**
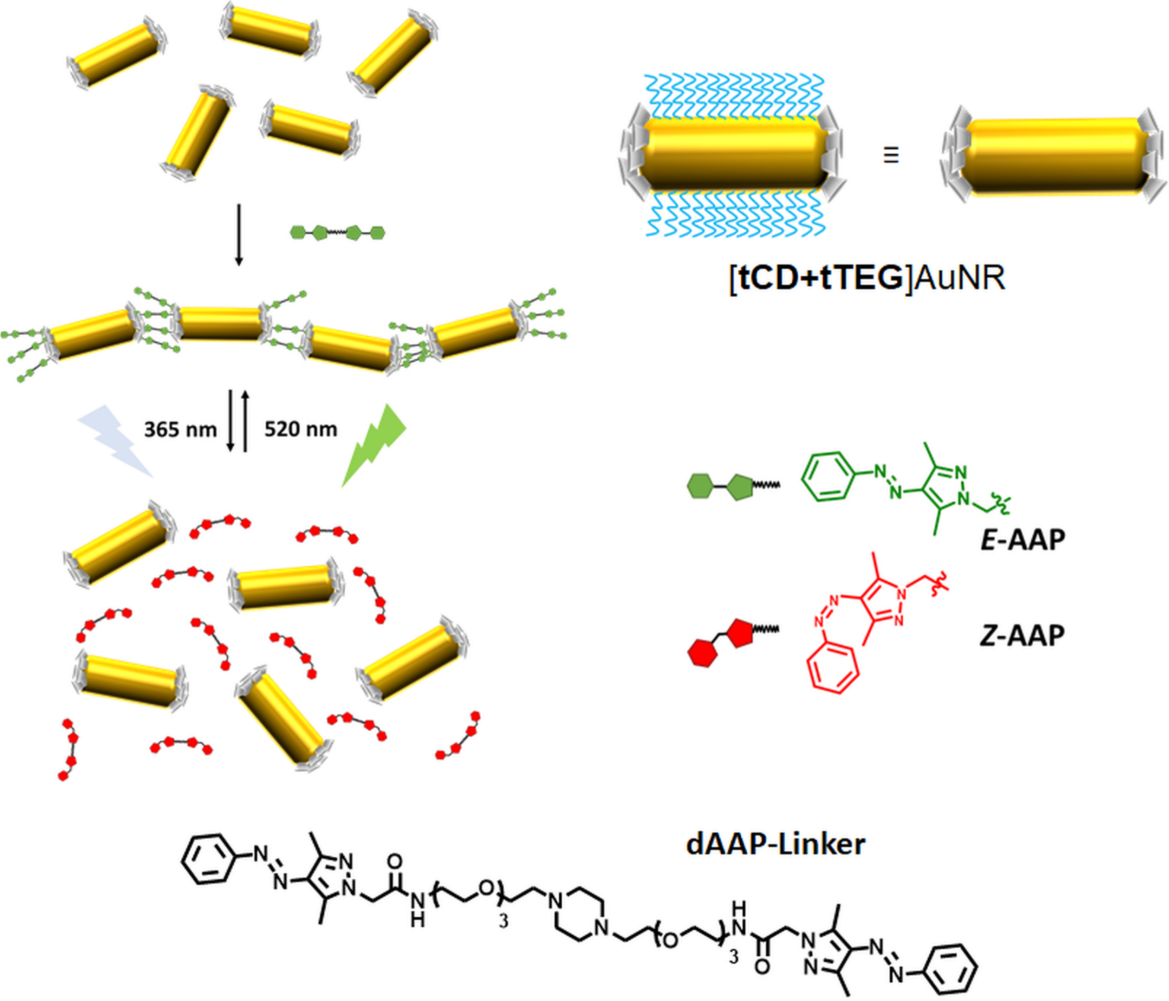
Light-responsive end-to-end assembly of host-functionalized gold nanorods (AuNR) by cyclodextrin–AAP interaction.

Herein, we present the application of a light-responsive CD–AAP host–guest system for the reversible end-to-end assembly of AuNR. The AuNR are functionalized selectively in a two-step ligand exchange reaction. The ends are capped with per-6-thio-β-cyclodextrin (tCD) and CTAB is removed from the remaining surface by monothiolated tetraethylene glycol (tTEG) enabling reversible host–guest chemistry. The addition of a divalent AAP linker molecule (dAAP) led to light-responsive reversible self-assembly of AuNR in an end-to-end manner as depicted in Scheme 1 to open up a novel strategy for the design of hybrid nanomaterials by supramolecular chemistry.

## Results and Discussion

**Scheme 2:**
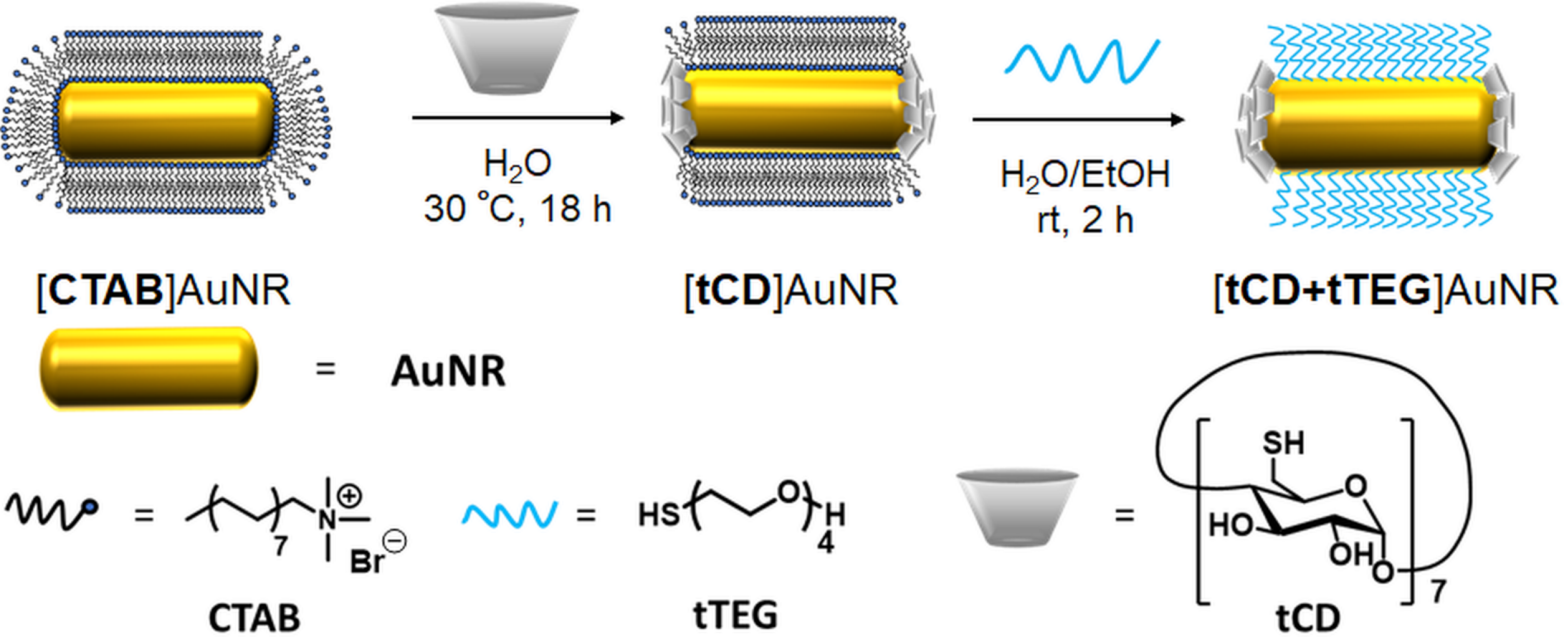
Two-step ligand exchange reaction for the synthesis of water-soluble cyclodextrin end-functionalized gold nanorods.

The AuNR (length: 58 nm, width: 25 nm, aspect-ratio: 2.3) were synthesized according to the seed-mediated growth procedure developed by Murphy [40] and El-Sayed [41]. CD-end functionalized AuNR were obtained in a two-step ligand exchange procedure as depicted in Scheme 2. For an efficient ligand exchange the primary alcohol groups of β-CD were replaced with thiols by a known two-step synthesis [42–43] to give a multivalent ligand (tCD) that is tightly bound to the gold surface and is not replaced by an excess of monothiolated tetraethylene glycol (tTEG). It is important to note the importance of the addition of ethanol during the ligand exchange to increase the solubility of CTAB and destabilize its double layer. Furthermore, ethanol weakens the hydrophobic effect of the solvent so that the inclusion of CTAB into β-CD is suppressed.

**Figure 1:**
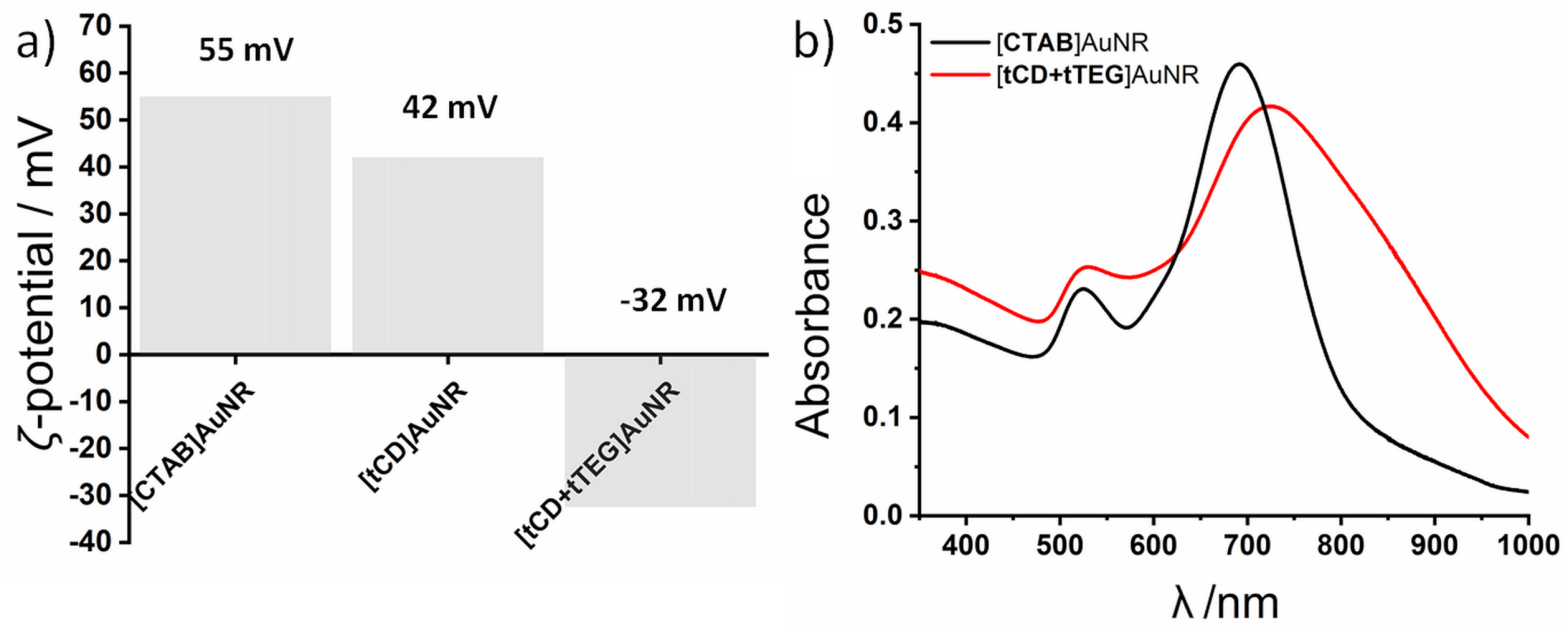
a) ζ-Potential measurement of different stages of the ligand exchange. b) UV–vis spectroscopy before (black) and after complete ligand exchange (red).

The ligand exchange was followed by ζ-potential measurements giving direct information about the surface charge potential and therefore about the capping agent (Figure 1a). CTAB-functionalized AuNR have a highly positive surface charge (+55 mV) due to the formation of CTAB double layers. The intermediate state [tCD]AuNR shows a decreased surface charge (+42 mV) indicating a reduction of the CTAB surface coverage. It is assumed by preliminary studies that the ligand exchange mainly emerges at the AuNR ends due to its high ligand exchange kinetics [10,15–16]. The addition of an excess of tTEG resulted in a negative surface charge (−32 mV) indicating a complete removal of CTAB from the gold surface. Furthermore, the high negative ζ-potential evidences electrostatic repulsion between the particles and thus good colloidal stability against uncontrolled aggregation. It is well known that PEGylated surfaces and particles show a negative ζ-potential in water due to the preferential absorption of hydroxide anions [44]. Furthermore, the ζ-potential is known to be highest in deionized water and highly pH-dependent [45].

The ligand exchange reaction was also analyzed by UV–vis spectroscopy (Figure 1b). The synthesized [CTAB]AuNR show the two expected absorption maxima at λ = 525 nm for the TSPR band and at λ = 690 nm for the LSPR band. After the ligand exchange the bands shift to λ = 528 nm TSPR and to λ = 721 nm LSPR accompanied by a general broadening of the SPR bands. This broadening can be explained by different chemical environments for diverse ligands and is a known characteristic for dual surfactant nanoparticle systems [46]. This interpretation is also supported by TEM images (Figure 4a, see below) indicating significant interparticle repulsion and no aggregation-induced absorption maxima broadening.

For further experiments the [tCD+tTEG]AuNR stock solution was diluted with ddH_2_O. For the photoresponsive aggregation of isotropic nanoparticles, the 1,4-bis(2-(2-(2-(2-aminoethoxy)ethoxy)ethoxy)ethyl)piperazine-based AAP linker molecule (dAAP) has been successfully applied before and it was synthesized as described in detail in the literature [38]. This divalent linker molecule can form a light-responsive 1:2 complex with host-functionalized AuNR as shown in Scheme 1. The light-responsive AAP moiety isomerizes upon irradiation with λ = 365 nm to the *Z*-state and upon irradiation with λ = 520 nm back to the *E*-state. A non-ionic linker design was chosen to eliminate the possibility that the assembly is affected by changes of the ionic strength.

**Figure 2:**
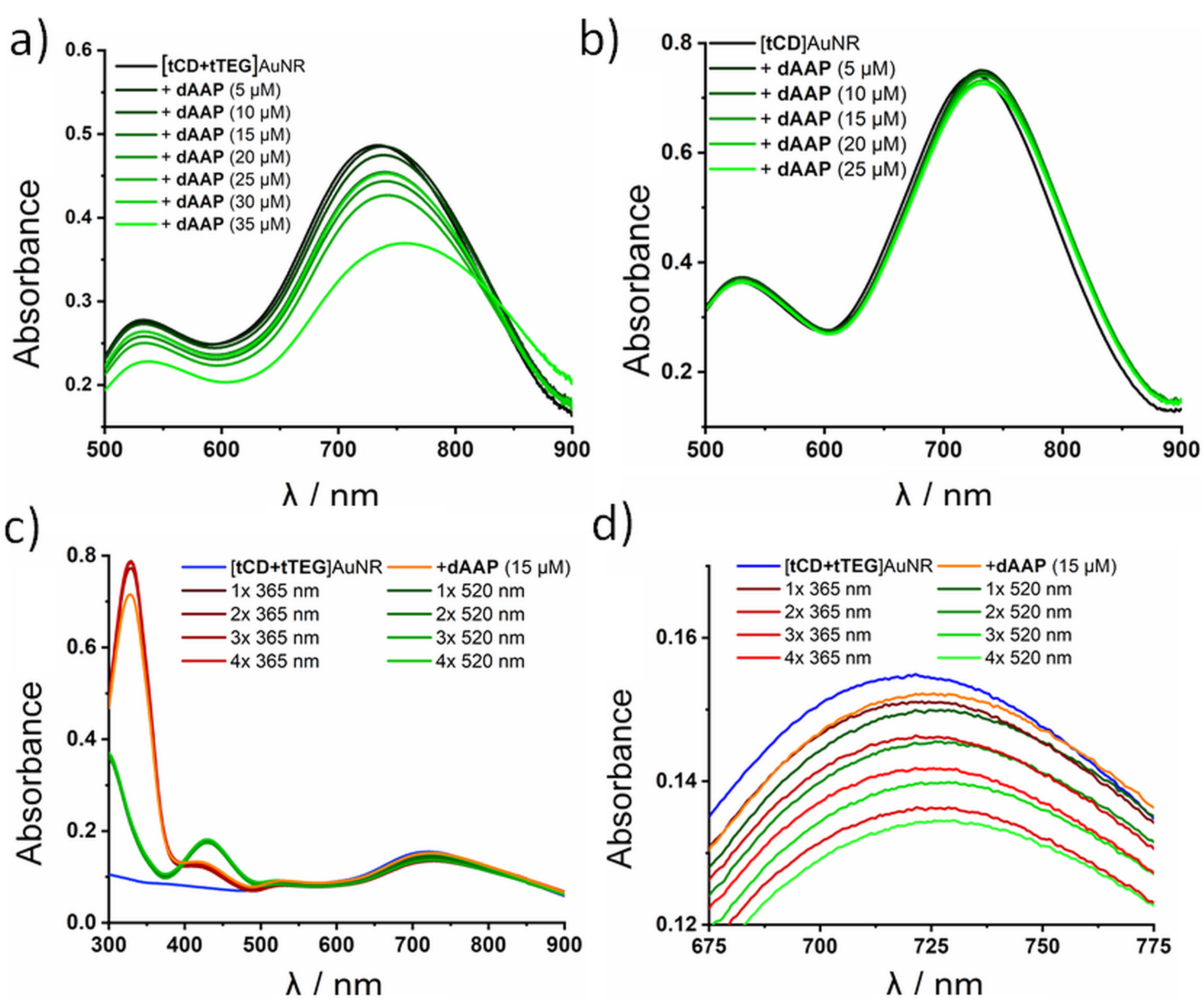
UV–vis spectroscopy of a) [tCD+tTEG]AuNR with different amount of dAAP (0-35 µM). b) [tCD]AuNR with dAAP (0–25 µM). c) [tCD+tTEG]AuNR with dAAP (15 µM). Assembly and disassembly by irradiation (5 min, 3 W lamp, λ = 360 nm and λ = 520 nm) over four cycles. d) Zoomed-in region of the LSPR.

Shortly after the addition of dAAP to the AuNR solution, UV–vis spectra were recorded (Figure 2a) indicating a strong LSPR shift of λ = 36 nm to λ = 757 nm while the TSPR band change is negligible. This indicates selective end-to-end assembly, whereas side-to-side or uncontrolled aggregation would result in an additional shift of the TSPR band [15]. In the course of repeated photoswitching experiments, the spectra show a small but significant decrease of the overall nanoparticle absorbance which is most likely due to sedimentation of irreversible aggregates or size-dependent nanoparticle settling and is in agreement with other studies on linear assemblies of AuNR [12]. To visualize the AuNR end-to-end assemblies, TEM measurements have been conducted (Figure 4b and c, see below) showing chains of AuNR. Hardly any unselective side-by-side or random assemblies can be observed which are often seen in various publications and are due to unspecific drying effects during the TEM grid preparation [11,27]. Adding the dAAP to the intermediate state of the ligand exchange ([tCD]AuNR, with CTAB surface coating on the sides of the particles) showed no significant shift of the SPR maxima (Figure 2b). This observation is consistent with the results of Ma et al. and reinforces the assumption that CTAB behaves as a competitive guest molecule towards β-CD. Therefore, reversible host–guest chemistry was not possible unless CTAB is completely removed.

**Figure 3:**
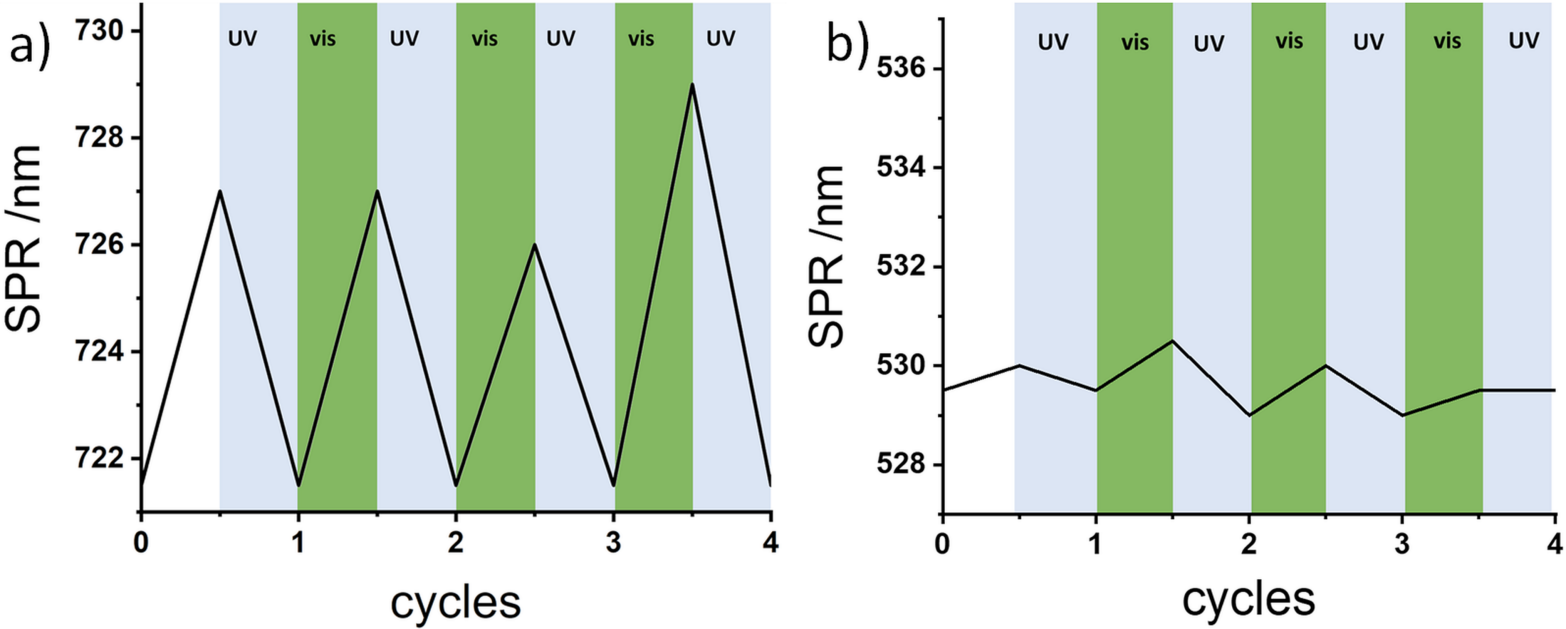
SPR maxima of [tCD+tTEG]AuNR with dAAP during four cycles of irradiation. a) Longitudinal SPR. b) Transversal SPR.

The reversibility of the light-responsive assembly was further investigated via UV–vis spectroscopy (Figure 2c and d). The spectrum measured after injection of dAAP shows a broadened LSPR maximum with a red shift of λ = 7 nm while the TSPR band remains unchanged. This indicates longitudinal coupling of AuNR as a result of the formation of end-to-end assemblies. Irradiation with UV light (λ = 365 nm, 5 min, 3 W) led to a sharpening of the LSPR band in addition to a blue-shifted maximum to λ = 721 nm, which is the same value recorded before the addition of dAAP. The LSPR and TSPR maxima have been plotted over four cycles showing only minor changes of the TSPR over the assembly (Figure 3). For reversible end-to-end assembly of AuNR, a shift λ < 10 nm is the expected range as it has been reported previously [18,38]. Longer irradiation times did not lead to stronger SPR band shifts and therefore all samples were irradiated for 5 min of the respective wavelength.

**Figure 4:**
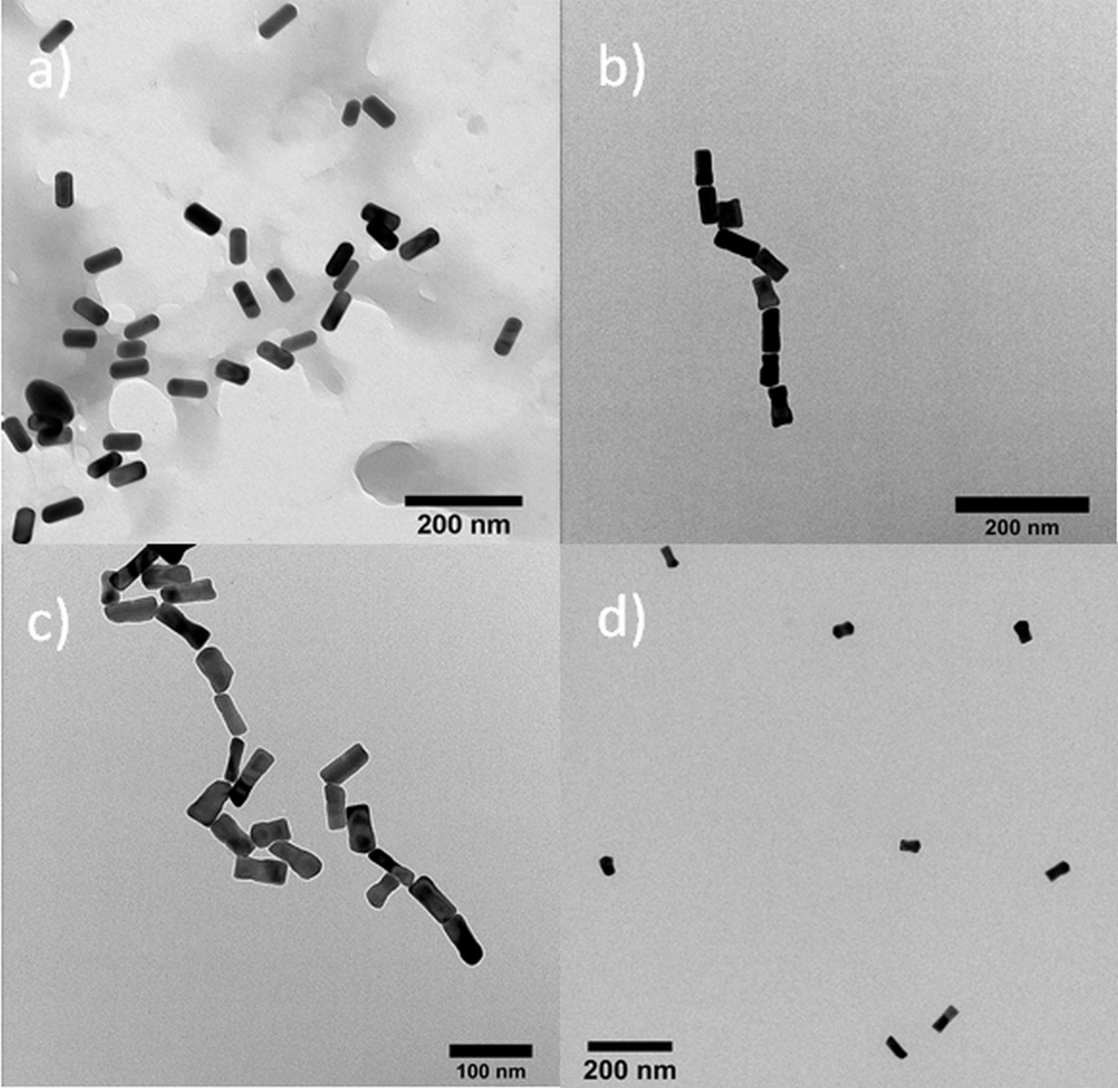
TEM-BF images of a) [tCD+tTEG]AuNR. b and c) AuNR end-to-end assemblies by dAAP (15 µM). d) Dissolved AuNR by UV irradiation (λ = 360 nm).

**Figure 5:**
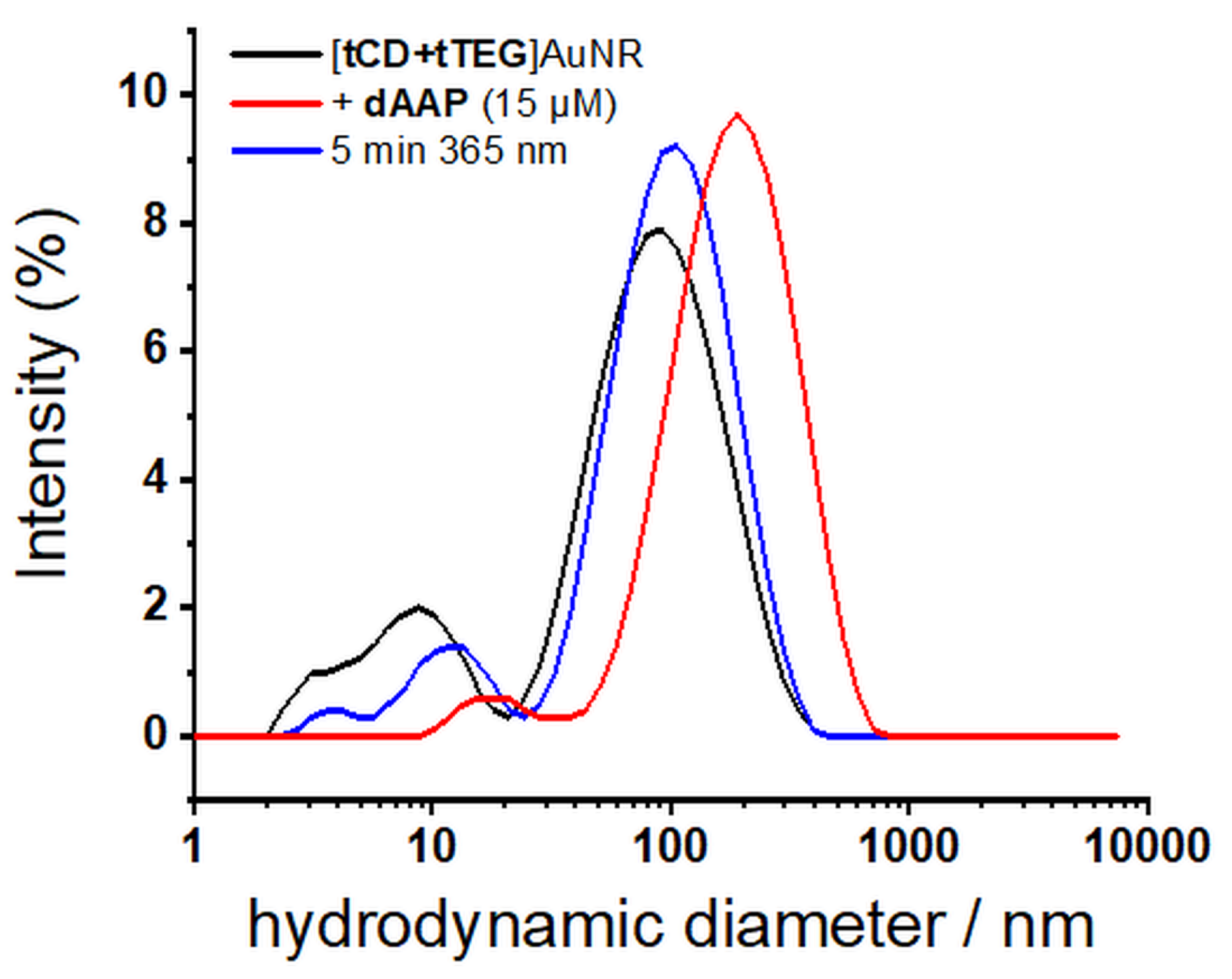
Reversible aggregation of [tCD+tTEG]AuNR by addition of dAAP (15 µM) monitored by dynamic light scattering.

Via irradiation with visible light (λ = 520 nm, 5 min, 3 W), the LSPR band is again broadened and shifted to a higher wavelength indicating the assembly of AuNR. This procedure could be repeated over four cycles without any fatigue effect and shows the good reversibility of the switching behavior. The dispersion of AuNR assemblies after visible light irradiation can also be verified by TEM bright field images (Figure 4d) showing that the disassembly is possible without the combination of irradiation and sonication. This can be attributed to the favorable photostationary states of the AAP moiety in comparison to azobenzenes used in previous studies. Furthermore, dynamic light scattering was conducted to analyze the assembly in solution (Figure 5). The mean diameter increases upon dAAP addition from ca. 100 nm to ca. 230 nm. Upon UV-light irradiation, the mean diameter decreases to 120 nm. We note that for anisotropic particles, the hydrodynamic diameter values give only a rough overestimation of the nanoparticle diameter and are not suitable to characterize end-to-end assembly. However dynamic light scattering clearly confirms the light-responsive aggregation of the AuNR [47].

Finally, to confirm that end-to-end assembly of AuNR occurs through the formation of a 2:1 complex of the [tCD+tTEG]AuNR and dAAP, a control experiment was performed by the addition of a monovalent AAP (mAAP) molecule (see Scheme S1 and Figure S3 in Supporting Information File 1). Even upon addition of rather high concentrations of mAAP (100 µM) no strong shift can be observed by UV–vis spectroscopy, indicating that supramolecular cross-linking is essential for end-to-end assembly of the AuNR.

## Conclusion

In summary, we reported a supramolecular system based on the reversible light-responsive interaction between arylazopyrazoles and β-CD for the end-to-end assembly of AuNR. The substitution of azobenzenes by AAP as guest molecules, which feature nearly quantitative photostationary states, allows the control over assembled and disassembled states solely by irradiation. The divalent AAP linker forms a 1:2 complex with per-6-thiolated β-CD which is selectively located at the ends of the AuNR. This was achieved through a novel two-step ligand exchange reaction including the complete removal of CTAB by the addition of thiolated TEG. The removal of CTAB is crucial due to its competitive interaction with β-CD since it has a higher binding constant than the AAP moieties. The success of the consecutive ligand exchange strategy was verified by ζ-potential measurements. The selective end-to-end assembly of AuNR was evidenced by UV–vis and TEM measurements. The LSPR shift could reversibly be shifted back and forth ending at the original wavelength. Switching of the end-to-end assembly was possible over four cycles without fatigue appearance. Previously reported limited feasibilities of azobenzenes could be resolved by the implementation of arylazopyrazoles. This report shows a successful method for the fabrication of new hybrid nanomaterials by the application of light-responsive host–guest chemistry allowing for effective control over assembled and disassembled states of anisotropic nanoparticles over several cycles.

## Experimental

### Instrumentation and materials

All chemicals were purchased from Sigma-Aldrich, Acros Organics, Merck, VWR or TCI and used without further purification if not stated otherwise. UV–vis absorbance measurements were performed with a V-770 double beam spectrophotometer (JASCO) at 25 °C. Samples for spectroscopic measurements were prepared in disposable 1 mL semi-micro PMMA cuvettes (BRAND). Transmission electron microscopy was performed using a Titan Themis G3 300 TEM (FEI) operating at 300 kV or a Libra 200 FE electron microscope (Zeiss) operating at 200 kV. The ζ-potential measurements were carried out on a Nano ZS Zetasizer (Malvern Instruments) at 25 °C and samples were prepared in disposable DTS 1060 capillary cells (Malvern Instruments). The syntheses of the tCD and tTEG can be found in Supporting Information File 1.

### AuNR seed-mediated growth

The CTAB-stabilized AuNR were synthesized according to a literature procedure [38–39]. All glassware used were rinsed with aqua regia before use. A 0.1 M aqueous solution of CTAB was prepared by gentle heating and sonication. Au seeds were prepared as follows: An aqueous solution of HAuCl_4_·3H_2_O (250 μL, 0.01 M) was added to an aqueous solution of CTAB (9.75 mL, 0.1 M) in a round-bottomed flask. The solution was stirred at 25 °C for 10 min. Then, a freshly prepared ice-cold aqueous solution of NaBH_4_ (600 μL, 0.01 M, prepared by diluting an 0.1 M solution) was added in one portion under vigorous stirring. After 10 min, stirring was slowed down to 200 rpm and continued at 25 °C. The seeds were kept at this temperature until their further use.

Growth of AuNR: Aqueous solutions of CTAB (95 mL, 0.1 M), HAuCl_4_·3H_2_O (5 mL, 0.01 M), AgNO_3_ (800 μL, 0.01 M) and ascorbic acid (800 μL, 0.1 M) were added in the given order to a 100 mL Erlenmeyer flask and were mixed after every addition by stirring. The addition of ascorbic acid turned the yellow solution colourless due to Au^3+^ to Au^+^ reduction. The flask was placed into a water bath of 25 °C. After 10 min, seed solution (120 μL) was added and mixed by gentle stirring. Stirring was continued for 18 h. The obtained AuNR solution was separated into two falcon tubes (50 mL) and centrifuged (6000 rpm, 20 min). The AuNR pellet was redispersed in H_2_O (50 mL each tube) and centrifuged again (6000 rpm, 20 min). The AuNR were dissolved in H_2_O stock solution of a total amount of 10 mL and stored in the fridge.

### [tCD+tTEG]AuNR ligand exchange

The isolated AuNR (1.9 mL of the stock solution) were diluted with H_2_O (8.1 mL) and tCD (3 mL, 0.5 mM, freshly prepared) was added dropwise under sonication. The solution was further sonicated for 10 min and then stirred for 18 h at 30 °C. A fresh aqueous solution of tTEG (2 mL, 10 mM) was added under sonication. The solution was stirred for 1 h at 30 °C. After this, EtOH abs. (3 mL) was added and stirring was continued at 30 °C for 1.5 h. DMSO (10 mL) was added and the resulting solution was centrifuged (6000 rpm, 10 min). The AuNR pellet was redispersed in H_2_O/EtOH 1:1 (10 mL) and centrifuged again (6000 rpm, 10 min). For final removal of excess ligands, the AuNR were purified by centrifugal filtration (2 × 3 mL H_2_O, 6000 rpm, 10 min, 10 kDa MWCO).

## Supporting Information

File 1 Experimental details and additional characterization data.
