# Supplementary material for: Reversible end-to-end assembly of selectively functionalized gold nanorods by light-responsive arylazopyrazole–cyclodextrin interaction
Source: Beilstein J Org Chem. 2019 Jun 26;15:1407–15. doi: 10.3762/bjoc.15.140 (PMC6604721; doi:10.3762/bjoc.15.140)
Supplement: File 1 — Experimental details and additional characterization data. [file Beilstein_J_Org_Chem-15-1407-s001.pdf]

BEILSTEIN JOURNAL OF ORGANIC CHEMISTRY

Supporting Information

for

Reversible end-to-end assembly of selectively functionalized gold nanorods by light-responsive arylazopyrazole–cyclodextrin interaction

Maximilian Niehues, Patricia Tegeder and Bart Jan Ravoo

Beilstein J. Org. Chem. **2019,** *15,* 1407–1415. doi:10.3762/bjoc.15.140

Experimental details and additional characterization data

License and Terms: This is a supporting information file under the terms of the Creative Commons Attribution License (http://creativecommons.org/licenses/by/4.0). Please note that the reuse, redistribution and reproduction in particular requires that the authors and source are credited.
The license is subject to the *Beilstein Journal of Organic Chemistry* terms and conditions: (https://www.beilstein-journals.org/bjoc)

Instrumentation and materials

Bright-field transmission electron microscopy (BF TEM) was performed on a Titan Themis G3 300 (FEI) operating at an accelerating voltage of 300 kV and a Libra 200 FE electron microscope (Zeiss) operating at 200 kV. Sample preparation was performed by incubation of a glow-discharged carbon-coated copper grid (S162, Plano) with 5 µL of the sample for 10 min followed by gentle blotting with filter paper. For the grid preparation of by UV light dissolved assemblies the sample (5 µL) was incubated for 10 min under irradiation (3 W, 365 nm) followed by blotting with filter paper. TEM images were analysed with TIA version 4.5 (FEI) and ImageJ version 1.52h (National Institutes of Health, USA, Java 1.8.0_66). Ultrapure water was obtained with a PureLab UHQ (ELGA LabWater) water purification system. ^{1}H NMR and ^{13}C NMR spectra were recorded on a DPX 300 (Bruker), Avance II 300 (Bruker) or an Avance II 400 (Bruker). Chemical shifts δ in ppm are referenced to the solvent residual peak. MALDI-MS were measured on an Autoflex Speed MALDI-TOF (Bruker). Irradiation experiments were performed with two different light sources with the power of 3 W. 365 nm UV irradiation was performed by Gen 2 Emitter UV LEDS (LED Engin). 520 nm green light irradiation was performed by LSC-G HighPower LEDs (Cree).

Experimental setup UV–vis titration

All assembly experiments were performed in ultrapure water. The nanoparticle stock solution was diluted to an UV–vis absorbance of the longitudinal SPR band of 0.5–0.7. For the titration experiments the total sample volume was 1 mL. dAAP was dissolved in DMSO (5 mM) and was added to the sample in amounts from 1–10 µL giving a total concentration from 0–50 µM. Two min after each addition a spectrum was recorded. For irradiation experiments, the sample cuvette was placed for 5 min in front of a light source of the respective wavelength (365 nm/520 nm) with the power of 3 W. For the switching experiments the solution was further diluted to a LSPR absorbance of 0.15 for better transmission. The UV–vis measurement was conducted immediately after irradiation. To avoid back isomerization the measurement room is tempered at 20 °C and kept dark.

Synthesis

*Per-6-iodo-β-*cyclodextrin [1]

β-Cyclodextrin (11.6 g. 10.2 mmol, 1.0 equiv) was dispersed in toluene and the solvent removed under reduced pressure. This procedure was repeated to remove residual water from the cavities. Next, the β-cyclodextrin was dried for 24 h at 50 °C under high vacuum. To a solution of triphenylphosphine (40.1 g, 153 mmol, 15 equiv) and iodine (40.5 g, 160 mmol, 15.7 equiv) in DMF (160 mL) the dried β-cyclodextrin was added under inert gas atmosphere and the solution was stirred at 80 °C for 18 h. The mixture was then concentrated under reduced pressure to half of the volume and the pH was adjusted to 9–10 by the addition of freshly prepared sodium methoxide in MeOH (3 M, 60 mL) with simultaneous cooling. The solution was stirred for 30 min at rt and afterwards poured into ice water (1.5 L). The precipitate was collected by filtration and washed with methanol. The brownish solid was purified by Soxhlet extraction with MeOH for 48 h. After drying, the desired product was obtained as white powder.

Molecular formula: $C_{42}H_{63}I_7O_{28}$.

Yield: 16.89 g (8.87 mmol, 87%)

^{1}H-NMR (300 MHz, DMSO-*d*6): *δ* = 6.06 (d, *J* = 6.2 Hz, 7H, OH-2), 5.94 (d, *J* = 2.1 Hz, 7H, OH-3), 4.98 (d, *J* = 3.5 Hz, 7H, H-1), 3.80 (d, *J* = 10.2 Hz, 7H, H-6b), 3.71 - 3.53 (m, 14H, H-3, H-5), 3.50-3.23 (m, 21H, H-2, H-4, H-6a) ppm.

^{13}C-NMR (101 MHz, DMSO-*d*6): *δ* = 102.6 (C-1), 86.4 (C-4), 72.7 / 72.4 (C-3, C-5), 71.5 (C-2), 10.0 (C-6) ppm.

MALDI-MS$^+$ (H2O/ACN): $[M+Na]^+$ calc.: *m/z* = 1926.67; exp.: 1926.68.

Per-6-thio-β-cyclodextrin (tCD) [2]

Per-6-iodo-β-cyclodextrin (4.17 g, 2.19 mmol, 1.0 equiv) was dissolved in DMF (43 mL) and thiourea (1.30 g, 17.08 mmol, 7.8 equiv) was then added and the reaction mixture heated to 70 °C under an inert gas atmosphere. After 19 h, the DMF was removed under reduced pressure to give a yellow oil, which was dissolved in H_2O (200 mL). Sodium hydroxide (1.12 g) was added and the reaction mixture was heated to reflux under an argon atmosphere. After 1 h, the resulting suspension was acidified with a saturated aqueous solution of $KHSO_4$ and the precipitate was filtered and washed thoroughly with distilled water. After drying under high vacuum, the desired product was obtained as white powder.

Molecular formula: $C_{42}H_{70}O_{28}S_7$.

Yield: 2.19 g (1.75 mmol, 80%).

^{1}H-NMR (300 MHz, DMSO-*d*6): δ = 5.93 (d, J = 6.9 Hz, 7H, 2-OH), 5.83 (s, 7H, 3-OH), 4.93 (d, *J* = 3.6 Hz, 7H, H-1), 3.75-3.55 (m, 14H, H-3, H-5), 3.21-3.34 (m, 21H, H-2, H-4, H6b), 2.86-2.63 (m, 7H, H-6a), 2.14 (t, *J* = 8 Hz, 7H, SH) ppm.

^{13}C-NMR (101 MHz, DMSO-*d*6): δ = 102.8 (C-1), 85.5 (C-4), 73.1 (C-2), 72.9 (C-3), 72.6 (C-5), 26.8 (C-6) ppm.

MALDI-MS$^+$ (EtOAc): $[M+Na]^+$ calc.: *m/z* = 1269.20; exp.: 1269.20.

Additional experimental data

Full UV–vis spectra:

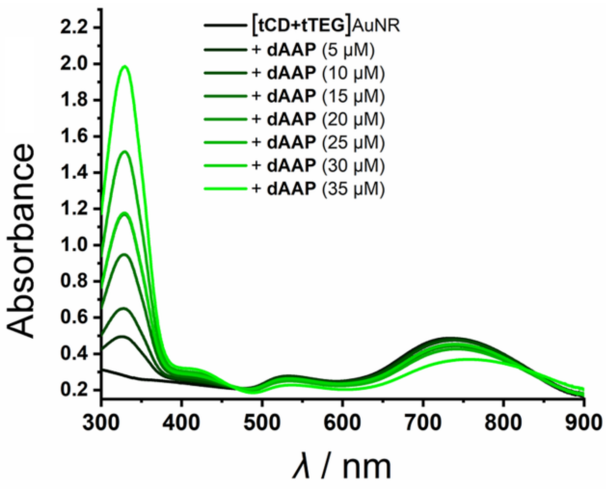

Figure S1: Full range UV–vis-spectrum of [**tCD+tTEG**]AuNR with the addition of dAAP (0–35 µM).

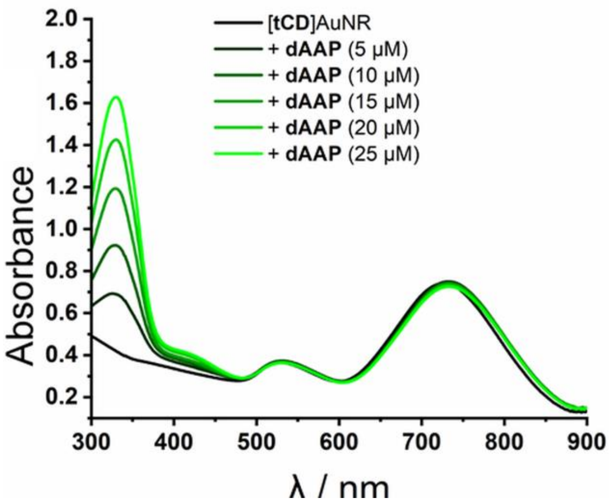

Figure S2: Full range UV–vis-spectrum of [**tCD**]AuNR with the addition of dAAP (0–25 µM).

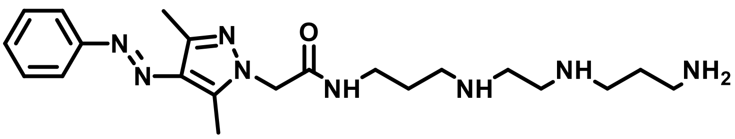

Scheme S1: Chemical structure of the monovalent AAP molecule. The synthetic procedure can be found in a previously reported study [3].

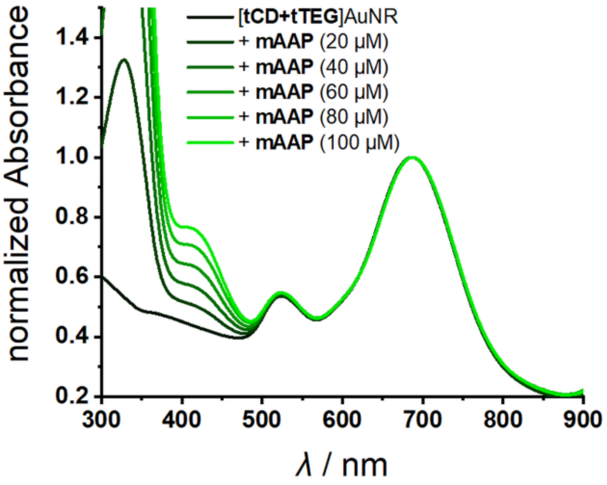

Figure S3: UV–vis spectrum of [**tCD+tTEG**]AuNR upon addition of mAAP (0–100 µM).

BF-TEM:

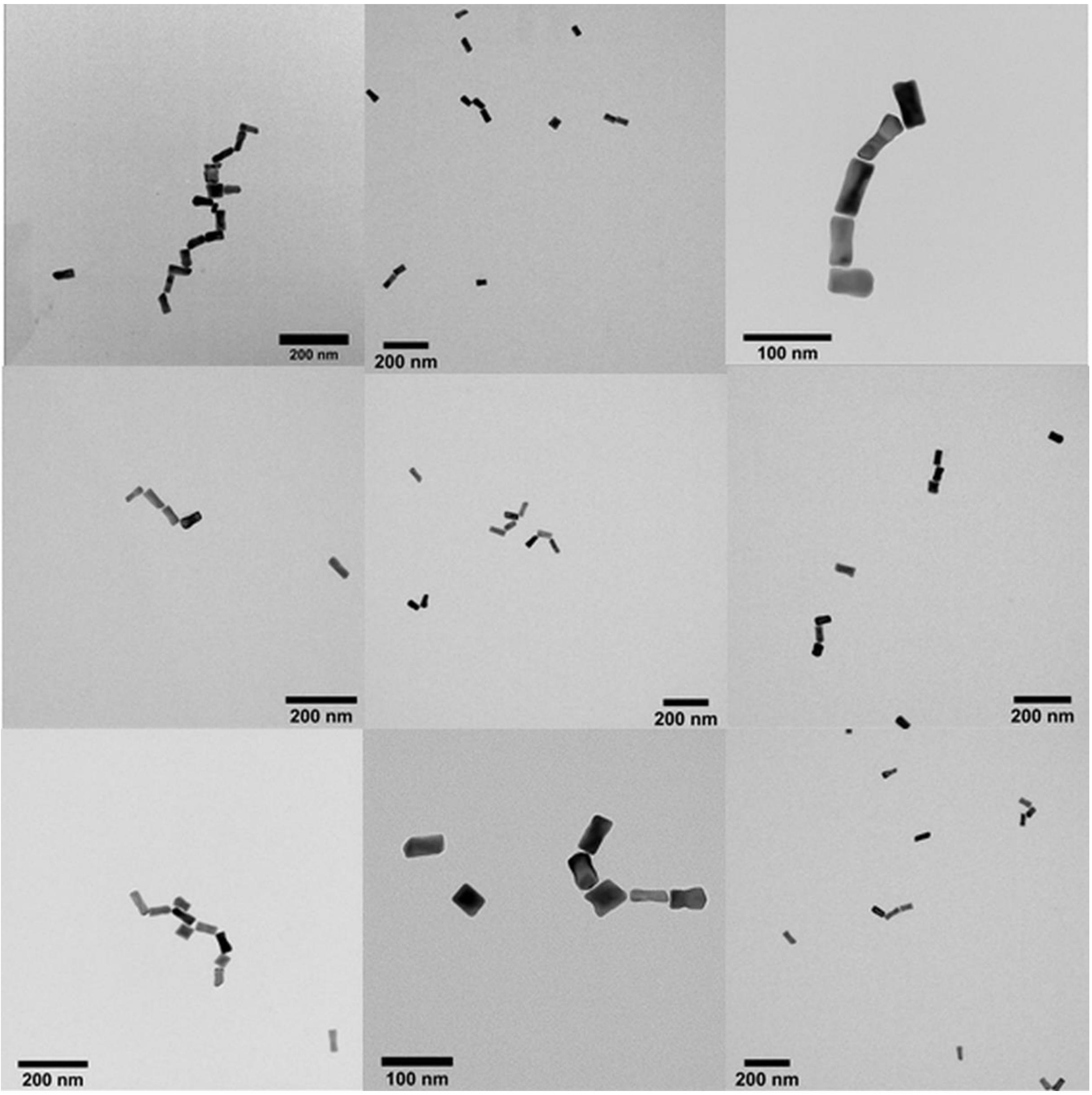

Figure S4: Additional BF-TEM images of end-to-end assemblies. [**tCD+tTEG**]AuNR + 15 µM dAAP.

Particle size distribution:

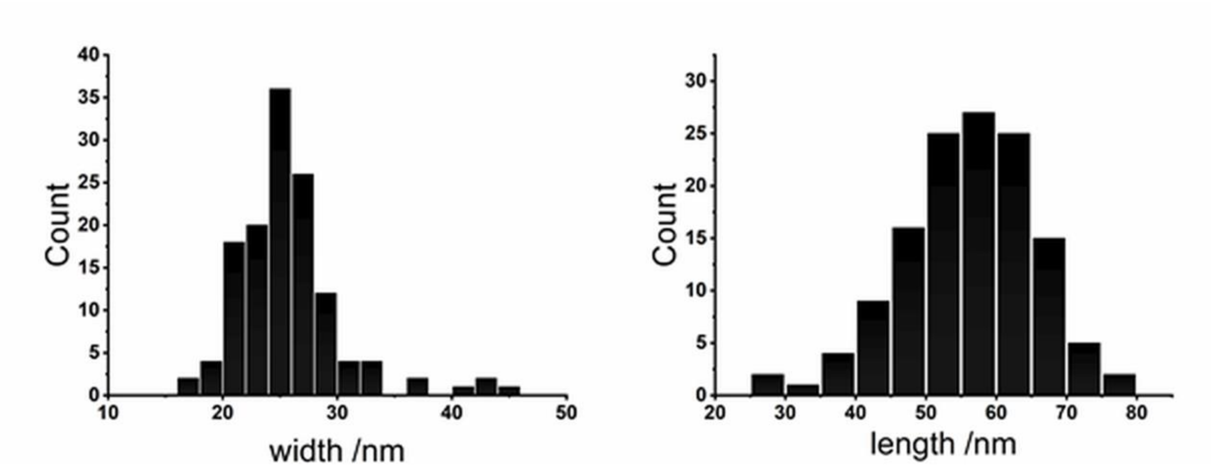

Figure S5: Left width, right length size distribution of [**CTAB**]AuNR. Total amount of counted particles = 120.

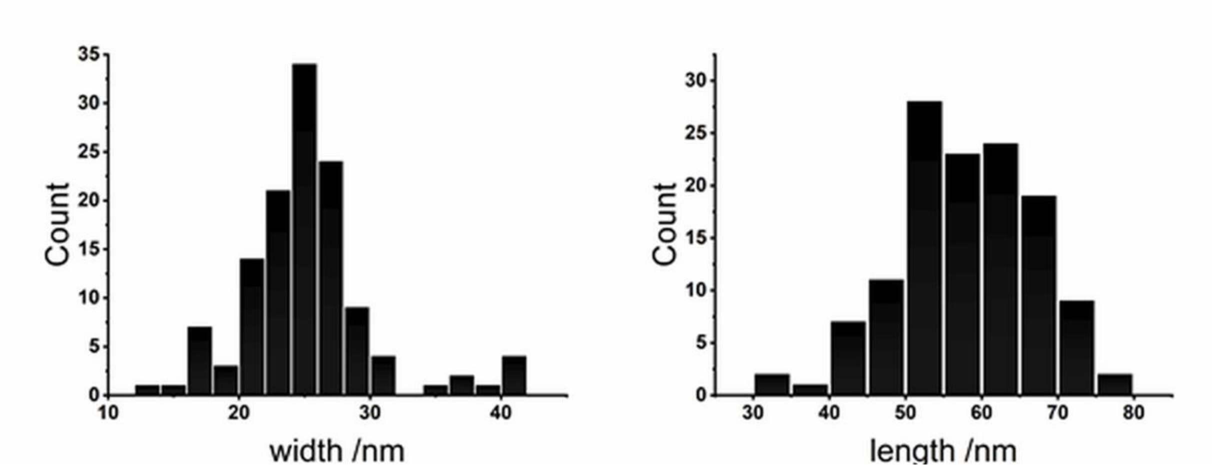

Figure S6: Left width, right length size distribution of [**tCD+tTEG**]AuNR. Total amount of counted particles = 120.

References

1. Gadelle, A.; Defaye, J. *Angew. Chem. Int. Ed.* **1991**, *30*, 78–80; *Angew. Chem*. **2011**, *123*, 9921–9925.

2. Rojas, M. T.; Kaifer, A. E.; Königer, R.; Stoddart, J. F. *J. Am. Chem. Soc.* **1995**, *117*, 336–343.

3. Stricker, L.; Fritz, E. C.; Peterlechner, M.; Doltsinis, N.; Ravoo, B. J. *J. Am. Chem. Soc.,* **2016**, *138*, 4547-4554.
